# Supplementary material for: Sharing brain mapping statistical results with the neuroimaging data model
Source: Sci Data. 2016 Dec 6;3:160102. doi: 10.1038/sdata.2016.102 (PMC5139675; doi:10.1038/sdata.2016.102)
Supplement: Supplementary File 1 [file sdata2016102-s1.pdf]

Checklist from (Poldrack et al. 2008)	NIDM-Results representation	Example (turtle)
	Intra-subject fMRI modeling info	
Estimation method	Attribute **nidm:'with Estimation Method'** of the **nidm:'Model Parameters Estimation'** activity.<br><br>Possible values include:<br>● obo:'ordinary least squares estimation' for ordinary least squares,<br>● obo:'generalized least squares estimation' for generalized least squares or;<br>● obo:'weighted least squares estimation' for weighted least squares.	`EXAMPLE: Ordinary Least Squares Estimation`<br>`@prefix nidm_ModelParametersEstimation: <http://purl.org/nidash/nidm#NIDM_0000056> .`<br>`@prefix nidm_withEstimationMethod: <http://purl.org/nidash/nidm#NIDM_0000134> .`<br>`@prefix obo_ordinaryleastsquaresestimation: <http://purl.obolibrary.org/obo/STATO_0000370> .`<br><br>`niiri:model_pe_id prov:used niiri:error_model_id ;`<br>`a prov:Activity , nidm_ModelParametersEstimation: ;`<br>`rdfs:label "Model parameters estimation" ;`<br>**`nidm_withEstimationMethod: obo_ordinaryleastsquaresestimation: .`**
Hemodynamic response function	Attribute **'has HRF Basis'** of a **'Design Matrix'** entity.<br><br>Possible values include:<br>● spm:'SPM's Canonical HRF' for SPM's canonical hemodynamic response function (default in SPM).<br>● fsl:'FSL's Gamma Difference HRF' for FSL's<br>● nidm:'Finite Impulse Response Basis Set'	`EXAMPLE: HRF: SPM's Informed Basis Set`<br>`@prefix nidm_DesignMatrix: <http://purl.org/nidash/nidm#NIDM_0000019> .`<br>`@prefix nidm_regressorNames: <http://purl.org/nidash/nidm#NIDM_0000021> .`<br>`@prefix nidm_hasHRFBasis: <http://purl.org/nidash/nidm#NIDM_0000102> .`<br>`@prefix nidm_hasDriftModel: <http://purl.org/nidash/nidm#NIDM_0000088> .`<br>`@prefix spm_SPMsCanonicalHRF: <http://purl.org/nidash/spm#SPM_0000004> .`<br>`@prefix spm_SPMsTemporalDerivative: <http://purl.org/nidash/spm#SPM_0000006> .`<br>`@prefix spm_SPMsDispersionDerivative: <http://purl.org/nidash/spm#SPM_0000003> .`<br><br>`niiri:first_level_design_matrix_id a prov:Entity , nidm_DesignMatrix: ;`<br>`rdfs:label "First-Level Design Matrix" ;`<br>`prov:atLocation "DesignMatrix.csv"^^xsd:anyURI ;`<br>`dct:format "text/csv"^^xsd:string ;`<br>`nfo:fileName "DesignMatrix.csv"^^xsd:string ;`<br>`dc:description niiri:design_matrix_png_id ;`<br>`nidm_regressorNames: "[\"Sn(1) active*bf(1)\",\"Sn(1) constant\"]"^^xsd:string ;`<br>`nidm_hasDriftModel: niiri:drift_model_id ;`<br>**`nidm_hasHRFBasis: spm_SPMsCanonicalHRF: ;`**<br>**`nidm_hasHRFBasis: spm_SPMsTemporalDerivative: ;`**<br>**`nidm_hasHRFBasis: spm_SPMsDispersionDerivative: .`**
Drift modeling/high-pass filtering	Attribute **'has Drift Model'** of a **'Design Matrix'** entity.<br><br>Possible values include:<br>● fsl:'Gaussian Running Line Drift Model' for a Gaussian-weighted running line smoother<br>● spm:'DCT Drift Model' for Discrete Cosine Transform basis.	`EXAMPLE: FSL's Gaussian Running Line Drift Model`<br>`@prefix fsl_GaussianRunningLineDriftModel: <http://purl.org/nidash/fsl#FSL_0000002> .`<br>`@prefix fsl_driftCutoffPeriod: <http://purl.org/nidash/fsl#FSL_0000004> .`<br><br>**`niiri:drift_model_id a prov:Entity , fsl_GaussianRunningLineDriftModel: ;`**<br>`rdfs:label "FSL's Gaussian Running Line Drift Model" ;`<br>`fsl_driftCutoffPeriod: "2"^^xsd:float .`
Autocorrelation model		

Model type	Attribute **'has Error Dependence'** of an **'Error Model'** entity.<br><br>Possible values include:<br>• obo:'Toeplitz covariance structure' for serially correlated error<br>• obo:'unstructured covariance structure' for arbitrary autocorrelation function	EXAMPLE: Error Model: SPM group analysis with non sphericity<br>@prefix nidm_ErrorModel: <http://purl.org/nidash/nidm#NIDM_0000023> .<br>@prefix nidm_hasErrorDistribution: <http://purl.org/nidash/nidm#NIDM_0000101> .<br>@prefix nidm_errorVarianceHomogeneous: <http://purl.org/nidash/nidm#NIDM_0000094> .<br>@prefix nidm_varianceMapWiseDependence: <http://purl.org/nidash/nidm#NIDM_0000126> .<br>@prefix nidm_hasErrorDependence: <http://purl.org/nidash/nidm#NIDM_0000100> .<br>@prefix nidm_dependenceMapWiseDependence: <http://purl.org/nidash/nidm#NIDM_0000089> .<br>@prefix nidm_IndependentParameter: <http://purl.org/nidash/nidm#NIDM_0000073> .<br>@prefix nidm_ConstantParameter: <http://purl.org/nidash/nidm#NIDM_0000072> .<br>@prefix obo_normaldistribution: <http://purl.obolibrary.org/obo/STATO_0000227> .<br>@prefix obo_unstructuredcovariancestructure:<br><http://purl.obolibrary.org/obo/STATO_0000405> .<br><br>niiri:error_model_id a prov:Entity , nidm_ErrorModel: ;<br>nidm_hasErrorDistribution: obo_normaldistribution: ;<br>nidm_errorVarianceHomogeneous: "false"^^xsd:boolean ;<br>nidm_varianceMapWiseDependence: nidm_IndependentParameter: ;<br>**nidm_hasErrorDependence: obo_unstructuredcovariancestructure: ;**<br>**nidm_dependenceMapWiseDependence: nidm_ConstantParameter: .**
Spatial definition	Attribute **'dependence Map-Wise Dependence'** of an **'Error Model'** entity.<br><br>Possible values include:<br>• 'Constant Parameter' for a global estimate.<br>• 'Independent Parameter' for a local estimate.<br>• 'Regularized Parameter' for a spatially regularized estimate.	
Contrast construction	Attribute **prov:value** of a **obo:'contrast weight matrix'** entity provides the contrast vector.	EXAMPLE: Contrast Weights<br>@prefix nidm_statisticType: <http://purl.org/nidash/nidm#NIDM_0000123> .<br>@prefix nidm_contrastName: <http://purl.org/nidash/nidm#NIDM_0000085> .<br>@prefix obo_contrastweightmatrix: <http://purl.obolibrary.org/obo/STATO_0000323> .<br>@prefix obo_tstatistic: <http://purl.obolibrary.org/obo/STATO_0000176> .<br><br>niiri:contrast_id a prov:Entity , obo_contrastweightmatrix: ;<br>rdfs:label "Contrast: Listening > Rest" ;<br>**prov:value "[1, 0, 0]"^^xsd:string ;**<br>nidm_statisticType: obo_tstatistic: ; # obo:'t-statistic'<br>nidm_contrastName: "listening > rest"^^xsd:string .
	Group modeling info	
Estimation method	(same as Intra-subject fMRI)	
	Statistical inference Inference on statistic image (thresholding)	
Search region for analysis<br><br>Location of the search space image	Attribute **prov:atLocation** of a **'Search Space Mask Map'** entity.	EXAMPLE: Search Space Mask Map<br>@prefix nidm_SearchSpaceMaskMap: <http://purl.org/nidash/nidm#NIDM_0000068> .<br>@prefix nidm_inCoordinateSpace: <http://purl.org/nidash/nidm#NIDM_0000104> .<br>@prefix nidm_expectedNumberOfVoxelsPerCluster:<br><http://purl.org/nidash/nidm#NIDM_0000143> .<br>@prefix nidm_expectedNumberOfClusters: <http://purl.org/nidash/nidm#NIDM_0000141> .<br>@prefix nidm_searchVolumeInVoxels: <http://purl.org/nidash/nidm#NIDM_0000121> .<br>@prefix nidm_searchVolumeInUnits: <http://purl.org/nidash/nidm#NIDM_0000136> .<br>@prefix nidm_reselSizeInVoxels: <http://purl.org/nidash/nidm#NIDM_0000148> .<br>@prefix nidm_searchVolumeInResels: <http://purl.org/nidash/nidm#NIDM_0000149> .

Volume of the search region in voxels.<br><br>Volume of the search region CC.	Attribute **'search Volume In Voxels'** of a **'Search Space Mask Map'** entity.<br><br>Attribute **'search Volume In Units'** of a **'Search Space Mask Map'** entity.	@prefix nidm_noiseFWHMInVoxels: <http://purl.org/nidash/nidm#NIDM_0000159> .<br>@prefix nidm_noiseFWHMInUnits: <http://purl.org/nidash/nidm#NIDM_0000157> .<br>@prefix nidm_randomFieldStationarity: <http://purl.org/nidash/nidm#NIDM_0000120> .<br><br>niiri:search_space_mask_id a prov:Entity , nidm_SearchSpaceMaskMap: ;<br>rdfs:label "Search Space Mask Map" ;<br>**prov:atLocation "SearchSpaceMask.nii.gz"^^xsd:anyURI ;**<br>nfo:fileName "SearchSpaceMask.nii.gz"^^xsd:string ;<br>dct:format "image/nifti"^^xsd:string ;<br>nidm_inCoordinateSpace: niiri:coordinate_space_id_2 ;<br>nidm_expectedNumberOfVoxelsPerCluster: "0.553331387916112"^^xsd:float ;<br>nidm_expectedNumberOfClusters: "0.0889172687960151"^^xsd:float ;<br>nidm_searchVolumeInVoxels: "65593"^^xsd:int ;<br>**nidm_searchVolumeInUnits: "1771011"^^xsd:float ;**<br>**nidm_reselSizeInVoxels: "22.9229643140043"^^xsd:float ;**<br>nidm_searchVolumeInResels: "2552.68032521656"^^xsd:float ;<br>nidm_noiseFWHMInVoxels: "[2.958, 2.966, 2.611]"^^xsd:string ;<br>nidm_noiseFWHMInUnits: "[8.876, 8.898, 7.835]"^^xsd:string ;<br>nidm_randomFieldStationarity: "true"^^xsd:boolean ;<br>crypto:sha512 "e43b6e01b0463fe7d40782137867a"^^xsd:string ;<br>prov:wasGeneratedBy niiri:inference_id .
Correction for multiple comparisons<br><br>Corrected or not?<br>Method used for correction<br><br>Region over which correction for multiple comparisons was performed	Attribute **prov:type** of the **'Height Threshold'** and the **'Extent Threshold'** used by an **'Inference'** activity<br><br>Possible values include:<br>● obo: 'FWER adjusted p-value' for an FWE-corrected threshold<br>● 'P-Value Uncorrected' for an uncorrected threshold<br>● obo:'q-value' for an FDR-corrected threshold<br><br>Attribute **prov:atLocation** of a **'Search Space Mask Map'** entity.	EXAMPLE: Voxel-wise p<0.05 FWER-corrected threshold<br>@prefix nidm_HeightThreshold: <http://purl.org/nidash/nidm#NIDM_0000034> .<br>@prefix nidm_equivalentThreshold: <http://purl.org/nidash/nidm#NIDM_0000161> .<br>@prefix obo_FWERadjustedpvalue: <http://purl.obolibrary.org/obo/OBI_0001265> .<br><br>niiri:inference_id prov:used niiri:height_threshold_fwer_id .<br><br>niiri:height_threshold_fwer_id a prov:Entity, nidm_HeightThreshold:,<br>**obo_FWERadjustedpvalue:** ;<br>rdfs:label "Height Threshold: p<0.05 (FWER-corrected)" ;<br>prov:value "0.05"^^xsd:float ;<br>nidm_equivalentThreshold: niiri:height_threshold_stat_id .
Voxel-wise significance<br><br>Corrected for Family-wise error (FWE) or false discovery rate (FDR)?<br><br>If FWE found by random field theory list the smoothness in mm FWHM<br><br>RESEL count	Attribute **prov:type** of the **'Height Threshold'** used by an **'Inference'** activity (cf. above for possible values),<br><br>Attribute **'noise FWHM In Units'** of a **'Search Space Mask Map'** entity.<br><br>Attribute **'search Volume In Resels'** of a **'Search Space Mask Map'** entity.	(cf. example for 'Search region for analysis' and 'Correction for multiple comparisons')

Cluster-wise significance		EXAMPLE: Cluster-wise p<0.05 FWER-corrected threshold with cluster-forming threshold of p<0.001 uncorrected<br>@prefix nidm_HeightThreshold: <http://purl.org/nidash/nidm#NIDM_0000034> .<br>@prefix nidm_PValueUncorrected: <http://purl.org/nidash/nidm#NIDM_0000160> .<br>@prefix nidm_ExtentThreshold: <http://purl.org/nidash/nidm#NIDM_0000026> .<br>@prefix obo_qvalue: <http://purl.obolibrary.org/obo/OBI_0001442> .<br><br>niiri:extent_threshold_fdr_id a prov:Entity, nidm_ExtentThreshold:, obo_qvalue: ;<br>rdfs:label "Extent Threshold: p<0.05 (FDR-corrected)" ;<br>prov:value "0.05"^^xsd:float .<br><br>niiri:height_threshold_unc_id a prov:Entity, nidm_HeightThreshold:, nidm_PValueUncorrected: ;<br>rdfs:label "Height Threshold: p<0.001 (uncorrected)" ;<br>prov:value "0.001"^^xsd:float .
cluster-defining threshold	Attribute **prov:value** of the '**Height Threshold'** used by an **'Inference'** activity.	
cluster significance level	Attribute **prov:value** of the **'Extent Threshold'** used by an **'Inference'** activity.	
smoothness (for random field theory)	Attribute **'noise FWHM In Units'** of a **'Search Space Mask Map'** entity.	
RESEL count	Attribute **'search Volume In Resels'** of a **'Search Space Mask Map'** entity.	

Supplementary File 1. Checklist to report neuroimaging results for intra-subject fMRI and group models from (Poldrack et al. 2008) and corresponding representation in NIDM-Results. The following items from the original checklist were excluded as not available automatically: “design type”, “orthogonalization of regressors”, “additional regressors used”,”if not whole brain, state how region of analysis was determined”, “If correction is limited to a small volume, the method for selecting the region should be stated explicitly”, “threshold used for visualization in figures”, “correction for multiple planned comparisons within each voxel”.
